# Tissue-Informative Mechanism for Wearable Non-invasive Continuous Blood Pressure Monitoring

**DOI:** 10.1038/srep06618

**Published:** 2014-10-21

**Authors:** Sung Hun Woo, Yun Young Choi, Dae Jung Kim, Franklin Bien, Jae Joon Kim

**Affiliations:** 1 UMEDIX Corporation Limited, Seoul, Republic of Korea; 2 School of Electrical and Computer Engineering, Ulsan National Institute of Science and Technology, Ulsan, Republic of Korea

## Abstract

Accurate continuous direct measurement of the blood pressure is currently available thru direct invasive methods via intravascular needles, and is mostly limited to use during surgical procedures or in the intensive care unit (ICU). Non-invasive methods that are mostly based on auscultation or cuff oscillometric principles do provide relatively accurate measurement of blood pressure. However, they mostly involve physical inconveniences such as pressure or stress on the human body. Here, we introduce a new non-invasive mechanism of tissue-informative measurement, where an experimental phenomenon called subcutaneous tissue pressure equilibrium is revealed and related for application in detection of absolute blood pressure. A prototype was experimentally verified to provide an absolute blood pressure measurement by wearing a watch-type measurement module that does not cause any discomfort. This work is supposed to contribute remarkably to the advancement of continuous non-invasive mobile devices for 24-7 daily-life ambulatory blood-pressure monitoring.

Blood pressure is the pressure exerted by the circulating blood against vessel walls, and is a vital sign in clinical assessments. It is used to clarify conditions of the cardiovascular system. For example, hypertension can result in strokes, heart attack, heart failure, aka ‘end-organ damage' phenomena^1^ that are critical factors leading to potential lethal conditions. Conversely, hypotension can cause dizziness, fainting, and endocrine or neurological disorders. Blood pressure is also crucial in other diseases such as diabetes mellitus. Lastly, blood pressure is also a useful indicator of normal cardiovascular conditions. With these considerations in mind, the continuous monitoring of blood pressure can lead to early discovery or at least immediate treatment of various cardiovascular diseases. Meanwhile, in cases of cardiovascular symptoms such as irregular heartbeat and arrhythmia, it is often difficult to discover a symptom with only a regular visit to the clinic. Moreover, at times during a clinic visit, blood pressure readings may be at variance from normal for psychological reasons, a condition known as ‘white-coat' hypertension. As such, there is considerable interest in continuous Ambulatory Blood Pressure Monitoring (ABPM) technology^2^ to continuously monitor blood pressure over the full course of a day.

Currently, the most accurate continuous blood pressure measurement is an invasive method^3^ that is used only for limited cases with critical needs, such as during surgery. In this invasive method, direct measurement of the blood pressure is possible through the electronic pressure transducer in an intravascular cannula needle inserted into a vein. However, due to potential complications such as thrombosis, infection, and bleeding^3^, even during a surgical procedure, the use of this invasive method is limited. As such, for daily monitoring purposes, a non-invasive ABPM method^4^ is much preferred over the invasive method. To date, ABPM devices are mostly based on auscultation^5^ or cuff oscillometry^6^ principles, both of which can be classified as occlusive technologies^7^ that use cuffs, and are non-continuous and originally devised by Korotkoff. Some drawbacks of these occlusive methods include petechiae, bruising, and even sleep disorders. As a result, this method is not recommended for use more than 5 to 8 times a day and would not be suitable for continuous blood pressure monitoring. Recently, semi-occlusive technologies have been developed to overcome the drawbacks of cuff-based occlusive technology. A volume clamp^8^ and tomometry^9^ are the most representative examples of these methods. These technologies, however, suffer from accuracy and reproducibility problems when tested against international standards^10^,^11^,^12^,^13^. The volume clamp method utilizes a cuff on a finger, while the tonometry approach requires sensors to have firm contact with the skin in order to touch a radial artery. These approaches continue to be insufficient for continuous ABPM functionality^14^,^15^,^16^.

As described above, there has been extensive research into continuous ABPM. Most previous approaches, however, came along with partial pain and non-continuity. In addition, the acquired waveform may have been proportional to the blood pressure while the methodology to acquire absolute blood pressure values was not fully developed. As such, in this paper, a new continuous blood pressure monitoring method, based on a newly discovered phenomenon and hypotheses, was proposed for continuous non-invasive ABPM (CNI-ABPM) that does not produce any discomfort or require the use of any cuffs. Through a prototype implementation and its measurement, the proposed method was verified to achieve meaningful accuracy correlation with respect to a validated commercial device.

## Results

### Principle of tissue-informative blood pressure detection

**Figure 1:**
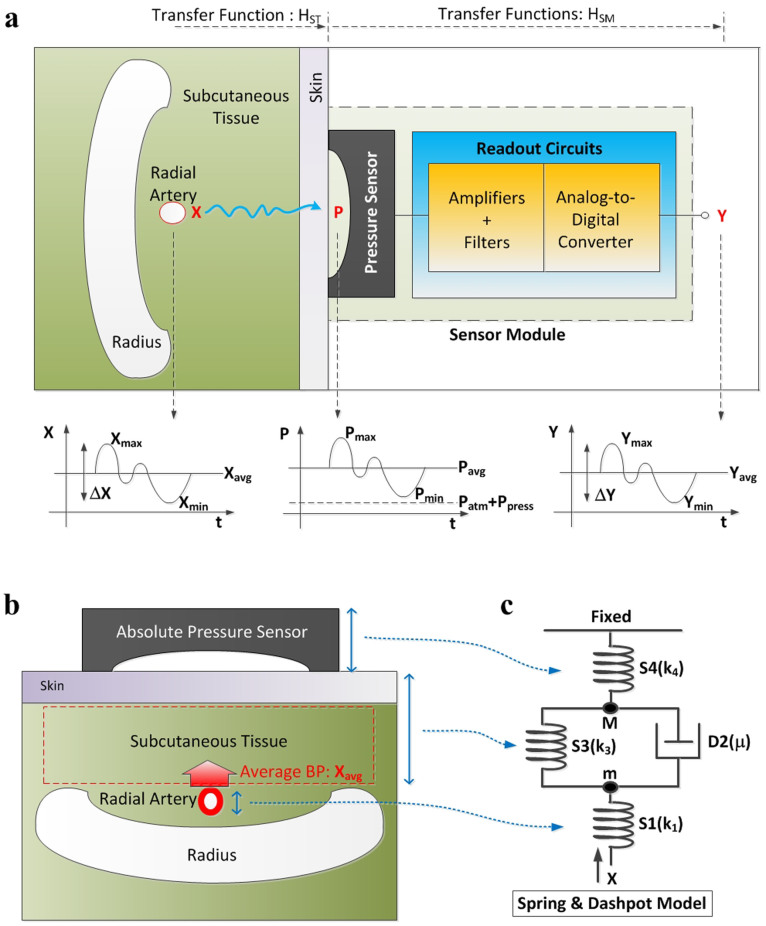
Principle of tissue-informative blood-pressure measurement. (a) Structural view of tissue-informative measurement in blood pressure (BP) of the radial artery. X is radial artery's pressure, P is pressure input to the pressure sensor, and Y is the final digitized output of the measurement module. (b) Vertical view from the skin to the radial artery. Subcutaneous tissue is sandwiched in-between the radial artery's blood pressure and external atmospheric pressure. (c) Spring and dashpot model to mimic the BP measurement.

To minimize the inconvenience of existing blood-pressure measurement methods, the proposed approach tries to simplify the acquisition process of human physiological signals and devises a new signal processing algorithm that can extract absolute blood pressure values from these measured physiological data. Our experimental environment is illustrated in Fig. 1, where the acquisition process is done by locating a pressure sensor onto the wrist near the radial artery. A pressure sensor translates the radial pulse (P) into a voltage signal, and a readout circuit filters out environmental noises and amplifies the desired signal, finally generating a digitized output of Y. The average value, Y_avg_ in Fig. 1a corresponds to the average pressure delivered to the skin, which is equal to the sum of the atmosphere pressure (P_atm_), external pressure applied by a measurement module (P_press_), and the average blood pressure delivered from the radial artery to the skin (H_ST_·X_avg_). Y's peak-to-peak amplitude, ΔY is normally proportional to the strength of the radial pulse wave. But with these values of ΔY and Y_avg_, it can measure only the external applied pressure P_press_ and the radial pulse wave delivered to the skin (Y), not the absolute blood pressure inside the radial artery (X).

To address this, we found an innovative experimental phenomenon, which we call “subcutaneous tissue pressure equilibrium (STPE),” that is applicable to absolute blood pressure detection. After observing radial skin dynamics with the radial artery under the atmosphere, we hypothesized that the radial skin with a radial artery of average blood pressure would be in an equilibrium state with atmospheric pressure. In addition, we further hypothesized that the pressure equilibrium would be sustained with the pressure sensor on it even under other pressure balance changes, since the subcutaneous tissue area (H_ST_) would be re-organized to balance the external pressure and the internal pressure from the average radial artery pressure. With these hypotheses, we tried the following experiments.

**Figure 2:**
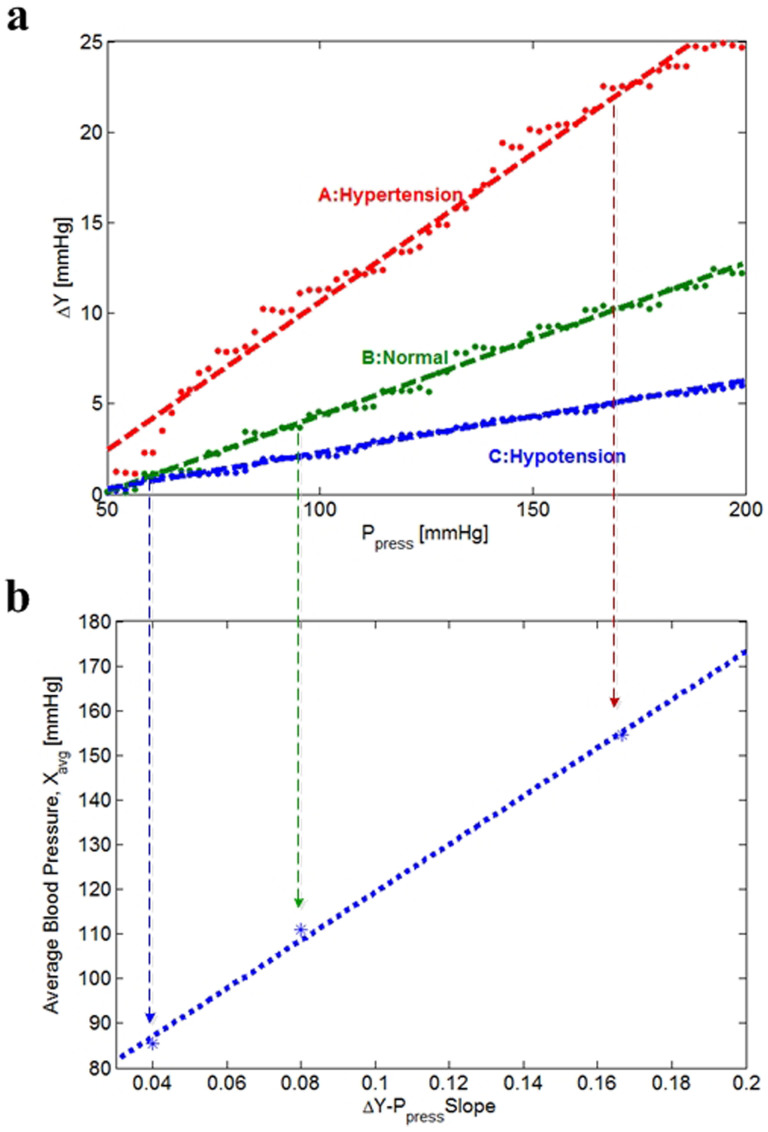
Experimental proofs of tissue-informative mechanism. (a) Measured ΔY-P_press_ characteristic curves depending on three different average blood pressures (A: hypertension, B: normal, C: hypotension). (b) Experimentally estimated relationship between X_avg_ and ΔY-P_press_ slope.

If the external applied pressure of P_press_ increases, the amplitude of the detected AC signal ΔY also increases proportionally, resulting in the straight line ΔY-P_press_ characteristic as shown in Fig. 2a. This is mainly due to the reduction of the propagation distance from the radial artery to the skin, i.e., a larger H_ST_. We found an important experimental phenomenon in that the slope of the ΔY-P_press_ line is proportional to the average blood pressure of the measured human, called the mean arterial pressure (X_avg_). That is, a higher X_avg_ gives a bigger slope, and a lower X_avg_ makes the slope smaller. This experimental proof is rearranged in Fig. 2b. This experimental result implies that if the subcutaneous tissue (H_ST_) area between the radial artery and the skin counteracts both the radial blood pressure and the atmospheric pressure for long time as in Fig. 1b, the transfer function of the tissue tends to be adaptively changed to acquire the other pressure equilibrium. In the case of hypertensive persons with higher X_avg_, their tissue has been exposed to a higher radial artery pressure, and the delivering characteristic of the radial pulse tends to be more sensitive, giving bigger slope of the ΔY-P_press_ line.

The STPE experimental phenomenon is theoretically analyzed by utilizing an intuitive modeling method proposed in Ref. 17. Fig. 1b shows a vertical view from the skin to the radial artery, and its equivalent spring and dashpot model is presented in Fig. 1c. Springs S1 and S4 model the radial artery and the pressure sensor with spring constants k_1_ and k_4_ representing their stiffness. The wall of the radial artery and the subcutaneous tissue are modeled as parallel configuration of a spring S3 and a dashpot D2. According to its mathematical analysis whose details are given in the supplementary method section, the stiffness of the tissue and the artery wall (k_3_) is directly related to their transfer efficiency of the original blood pressure. If this analysis result is combined with the experimental phenomenon in Fig. 2, it concludes that higher X_avg_ increases the k_3_ value and thereby improves the transfer efficiency, which results in bigger line slope of the ΔY-P_press_ characteristic.

### Recursive tracking of blood pressure

**Figure 3:**
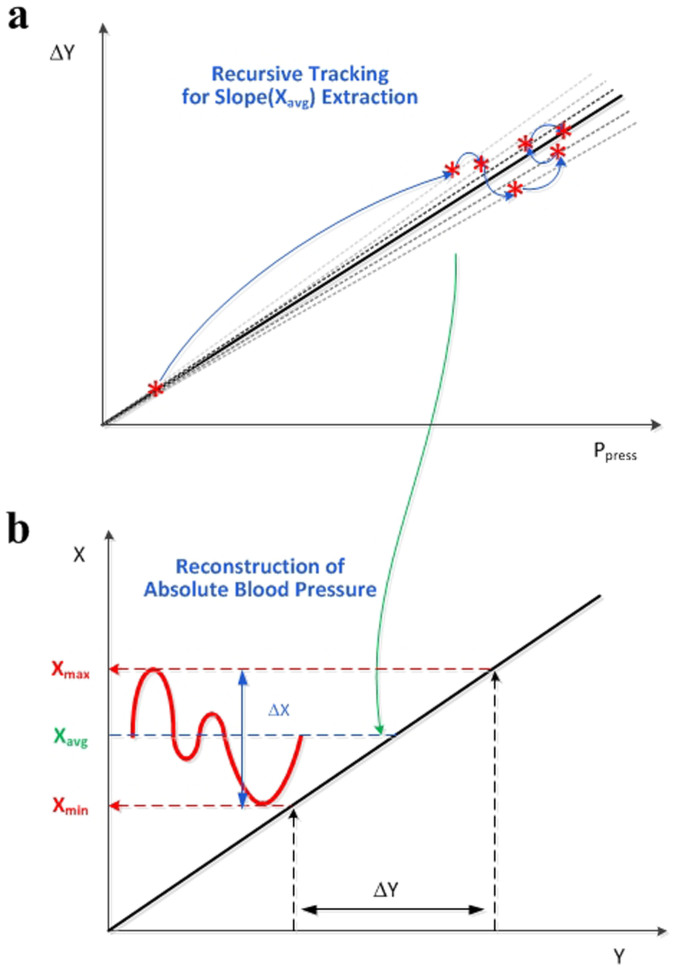
Recursive tracking of blood-pressure measurement. (a) Real-time slope tracking process in ΔY versus P_press_ plot. The line slope is cumulatively updated by last measured value. (b) Reconstruction process of radial artery's blood pressure (i.e., systolic pressure X_max_ and diastolic pressure X_min_) corresponding to updated X_avg_ which is calculated from ΔY-P_press_ line slope.

Based on this tissue-informative detection principle, an average blood pressure X_avg_ can be found by measuring ΔY variations with respect to various P_press_ values, that is, the ΔY-P_press_ slope. Only if the slope and X_avg_ are measured, the peak-to-peak value of the absolute blood pressure (ΔX) can be detected following the calculation process shown in Fig. S1. Since the average and the peak-to-peak value are directly related with the maximum and the minimum values, the systolic pressure (X_max_) and the diastolic pressure (X_min_) are easily derived from the measured values of X_avg_ and ΔX. Nevertheless, human blood pressure undergoes many instant variations, called human artifacts, and environmental atmospheric pressure also easily changes. Therefore, these instant variations necessitate a kind of tracking mechanism for better measurement. Fig. 3 shows the concept of a proposed real-time measurement algorithm, called “recursive tracking”.

After wearing our watch-type measurement device as shown in Fig. S2b, the average blood pressure of X_avg_ is cumulatively updated depending on instant small variations of P_press_, and then the resulting systolic and diastolic pressures are also cumulatively adapted. Unlike existing occlusive measurement devices that add an uncomfortable physical pressure on the body, this recursive tracking method allows continuous absolute blood pressure measurements and enables most comfortable solution for the CNI-ABPM.

### Continuous non-invasive ambulatory blood pressure monitoring

An overall system and its service scheme is illustrated in Fig. S2a, where the CNI-ABPM function is compactly implemented as a type of watch device and wireless technologies such as Bluetooth and cellular communications are included to maximize portability. The watch-type CNI-ABPM module acquires the radial pulse wave and its digitized information is transferred to a smart phone. Then the absolute blood pressure acquisition and recursive tracking functions are performed by utilizing a microprocessor inside the smart phone. Acquired blood pressure information, including the systolic and the diastolic pressure, is displayed on the smart phone and also can be delivered to medical institutions through a cellular network and the Internet. By transferring the processing burden of the pressure acquisition and the recursive tracking from the module to the smart phone, the CNI-ABPM module could be miniaturized and also very comfortable to wear during 24-hour monitoring. Fig. S2b shows a prototype of the CNI-ABPM module, which mainly consists of a pressure sensor, readout circuits, and Bluetooth module. The pressure sensor measures the physical pressure amount and generates its corresponding voltage. This voltage signal is amplified and digitized in the readout circuits. Then this acquired digital sensor information is wirelessly transmitted to the smart phone though the Bluetooth module. A bubble cap is located between the skin and the pressure sensor to improve the transfer efficiency of the radial pulse wave and to also make wearing of the device more comfortable.

**Figure 4:**
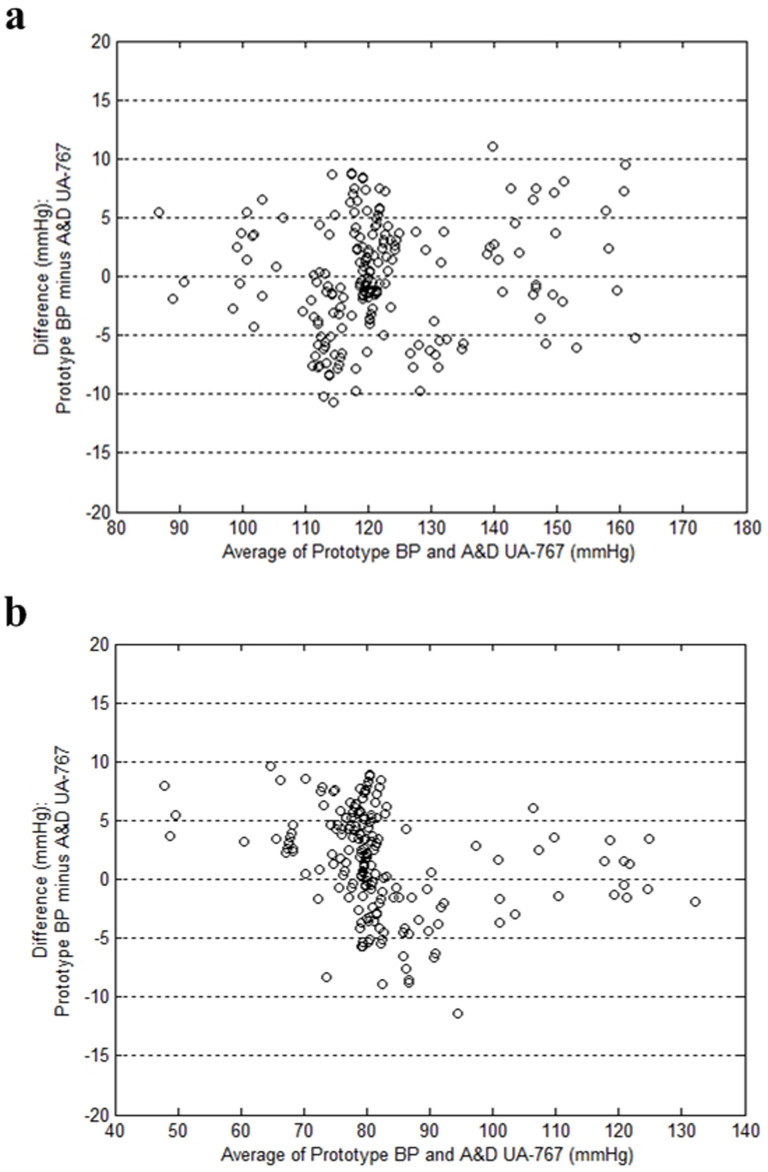
Measured results for the accuracy validation. Readings from 200 human subjects were analyzed to compare the prototype device with the previously-validated commercial product UA-767 of A&D Medical. (a) Bland-Altman Plot for systolic blood pressure. (b) Bland-Altman Plot for diastolic blood pressure.

International validation standards from the British Hypertension Society^18^ (BHS), the Association for the Advancement of Medical Instrumentation^19^,^20^ (AAMI), and the Working Group on Blood Pressure Monitoring of the European Society of Hypertension^21^,^22^,^23^,^24^ (ESH) should follow strict procedures and also require well-trained experts for manual sphygmomanometers. Moreover, the gold standard for continuous blood pressure measurement, the invasive intravascular method requires expensive surgical actions with potential complications^25^. Therefore, for accuracy verification of the proposed CNI-ABPM mechanism, a brief measurement and analysis method of randomized crossover^25^ with an automated validated device as the reference was adopted to collect many experimental data effectively. The implemented prototype device was compared with a commercial non-invasive automated oscillometric product UA-767 from A&D Medical which is validated with protocols set by organization such as the BHS, the AAMI, and the ESH. Two hundred adults are included in this crossover measurement. Measured Bland-Altman plots^25^ which graphically represent limits of agreements are given in Fig. 4. In case of the systolic blood pressure (SBP), its mean difference and standard deviation was 1.1 mmHg and 4.7 mmHg respectively. The diastolic blood pressure (DBP) showed mean difference of −1.9 mmHg and standard deviation of 4.4 mmHg. The recruited subjects presented the SBP range of 87–164 mmHg and the DBP range of 48–133 mmHg. Considering that the measured crossover experimental results are comparable to the AAMI accuracy criteria which are mean value of less than 5 mmHg and standard deviation of less than 8 mmHg, the crossover experiment on the accuracy in blood pressure measurement shows close correlation between the proposed device and the validated commercial product.

## Discussion

Many measurement technologies to monitor radial pulse waves utilize simple contact of pressure sensors to the skin, but they do not measure absolute blood pressures. Most validated methods that provide absolute pressures correspond to occlusive technology using a cuff, and they are fundamentally based on Korotkoff's sound detection or cuff oscillometry which are not appropriate for continuous ABPM. Among advanced semi-occlusive technologies that reduce the inconvenience of the cuff, volume clamp technology is supposed to be the only unsupervised method for continuous non-invasive blood pressure measurement. It is recognized now, however, that this does not fulfill the BHS nor AAMI standard criteria. Recently a watch-type measurement device using tonometry technology was announced^27^. This device, however, requires periodic calibrations to other cuff-based oscillometric device for accuracy compensation, and may also give relatively high pressure reading or add stress on the skin during the tonometry operation. Whereas, our proposed technology does not create any inconvenience or stress and it provides comfortable blood pressure measurement to facilitate 24-hour continuous non-invasive ABPM.

The STPE phenomenon and the proposed tissue-sensitive mechanism were theoretically modeled and analyzed through the spring and dashpot model which is adopted from a radial-artery tonometry model^17^ thanks to their similar physical structures. The analysis result revealed that the absolute blood pressure measurement can be achieved by utilizing the STPE phenomenon while the tonometry can detect only relative blood pressure signals delivered from the radial artery. The proposed tissue-informative detection method based on this STPE phenomenon was implemented in the form of app software programming inside smart phones, which minimizes the hardware burden of the sensor module and makes very small and comfortable to wear. Another important implementation technology is a measurement tracking function. To acquire the absolute blood pressure value, our detection method utilizes external pressure from the module to the skin which varies continuously. Whereas most other products use a cuff for similar purposes, our prototype adopted a recursive-tracking algorithm that was also implemented in the app software. As such, our blood pressure module was able to eliminate the cuff and its associated inconveniences that would limit continuous ABPM.

## Methods

### Absolute blood pressure detection

An implemented watch-type CNI-ABPM module acquires an instant radial pulse signal through a pressure sensor and readout circuits and sends it to a smart phone. Based on this real-time measured radial pulse data, the ΔY-P_press_ curve of Fig. 3 is constructed continuously. As in Fig. S1, the relationship between its slope and average blood pressure (X_avg_) is given by X_avg_ = (Slope/H_SM_ΔX_pre_ − H_ST0_)/k, and the peak-to-peak value of blood pressure (ΔX) is ΔX = ΔY /(H_ST0_ + kX_avg_)H_SM_ (P_press_ + P_atm_), where ΔX_pre_ is previous value of ΔX in the recursive algorithm and k is a constant value experimentally determined. These detection procedures and relationships are implemented in the form of app software on a smart phone, resulting in real-time continuous measurement of absolute blood pressure.

### Extraction of systolic and diastolic blood pressures

According to the proposed blood pressure measurement procedure, the average (X_avg_) and the peak-to-peak value (ΔX = X_max_ − X_min_) are found first. Then systolic pressure (X_max_) and diastolic pressure (X_min_) are calculated by using the relationship of X_avg_ = (αX_max_ + βX_min_)/(α + β). That is, X_max_ = X_avg_ + βΔX/(α + β) and X_min_ = X_avg_ − αΔX/(α + β), where α = 1 and β = 2 for normal blood pressure. This calculation is also implemented inside the smart phone.

### Accuracy verification

Commercial blood pressure devices are usually validated with protocols set by international standards such as the BHS, the AAMI, and the ESH. Both the BHS and the ESH validates the accuracy grade of commercial devices, and their grading criteria are composed of cumulative percentage of difference readings falling within 5 mmHg, 10 mmHg, and 15 mmHg^18^,^21^. The AAMI suggests statistical criteria that the mean difference and its standard deviation should be less than 5 mmHg and 8 mmHg respectively^19^,^20^. Since this work pursues accuracy verification of a new detection method, not validation of commercial devices, the accuracy correlation with other validated commercial device is performed. As the correlation standard, the AAMI criteria were adopted because it is more intuitive and convenient for accuracy comparison between two different measurement devices. A prototype device based on the proposed mechanism is compared with an automated oscillometric commercial device UA-767, and their accuracy correlation is investigated whether it meets the AAMI criteria or not. This study was approved by the institutional review board at Ulsan National Institute of Science and Technology, and all experiments were performed in accordance with the approved guidelines. Two hundred adults were recruited in the crossover measurement, and informed consent was obtained from all subjects. Every subject is seated with legs uncrossed and arm supported at heart level, and two measurements per each device are performed. If two measured values are not correlated, another measurement is made. Then, the average of two correlated measurements is recorded as the measured result. Finally, the correlation between two devices is evaluated by the mean difference and its standard deviation, and graphically presented with Bland-Altman plots of the SBP and the DBP where the x-axis is the average of measured blood pressures by two devices and the y-axis is their corresponding difference^26^.

## Author Contributions

S.H.W., Y.Y.C. and J.J.K. conceived and verified the concept and analyzed experimental results. Y.Y.C. and D.J.K. have contributed to development of the software. Y.Y.C., D.J.K., S.H.W., F.B. and J.J.K. have contributed to implementation of the hardware. J.J.K., F.B. and S.H.W. wrote the paper.

## Supplementary Material

Supplementary Information Supplementary Information

Supplementary Information Supplementary Video
