# Supplementary material for: Tissue-Informative Mechanism for Wearable Non-invasive Continuous Blood Pressure Monitoring
Source: Sci Rep. 2014 Oct 21;4:6618. doi: 10.1038/srep06618 (PMC4204067; doi:10.1038/srep06618)
Supplement: Supplementary Information [file srep06618-s1.pdf]

Supplementary Information

Tissue-Informative Mechanism for Wearable Non-invasive Continuous Blood Pressure Monitoring

Sung Hun Woo[1], Yun Young Choi[1], Dae Jung Kim[1], Franklin Bien[2] & Jae Joon Kim[2,*]

[1]UMEDIX Corporation Limited, Seoul, Republic of Korea.

[2]School of Electrical and Computer Engineering, Ulsan National Institute of Science and Technology, Ulsan, Republic of Korea

Correspondence and requests for materials should be addressed to J.J.K. (email: jaejoon@unist.ac.kr)

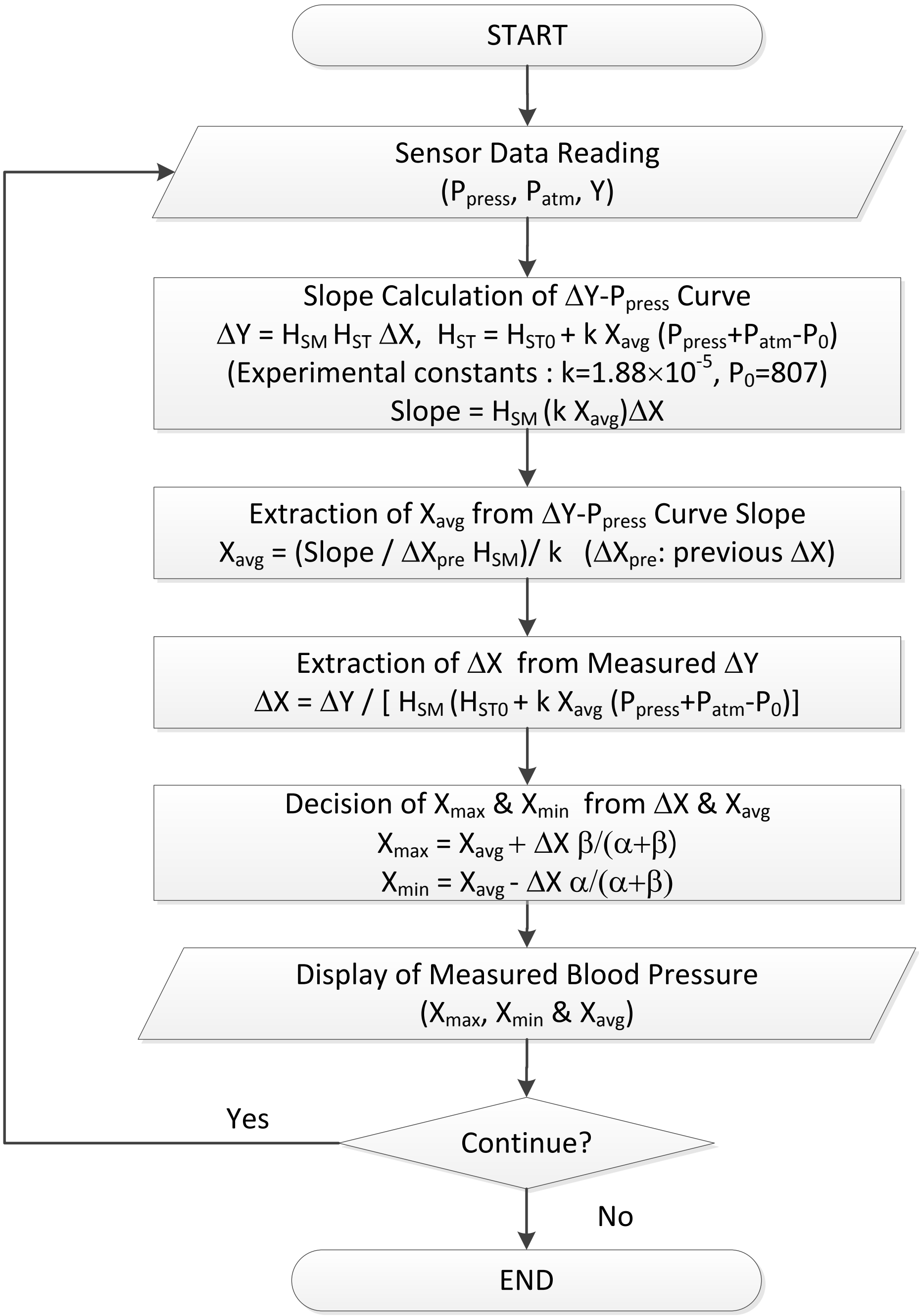

Supplementary Figure S1 | Measurement Process of Blood Pressure. Measurement process begins with data reading of a pressure sensor. Through five intermediate process, absolute blood pressure information including average value (X_{avg}), systolic pressure (X_{max}), and diastolic pressure (X_{min}) is acquired and then displayed on a smart phone.

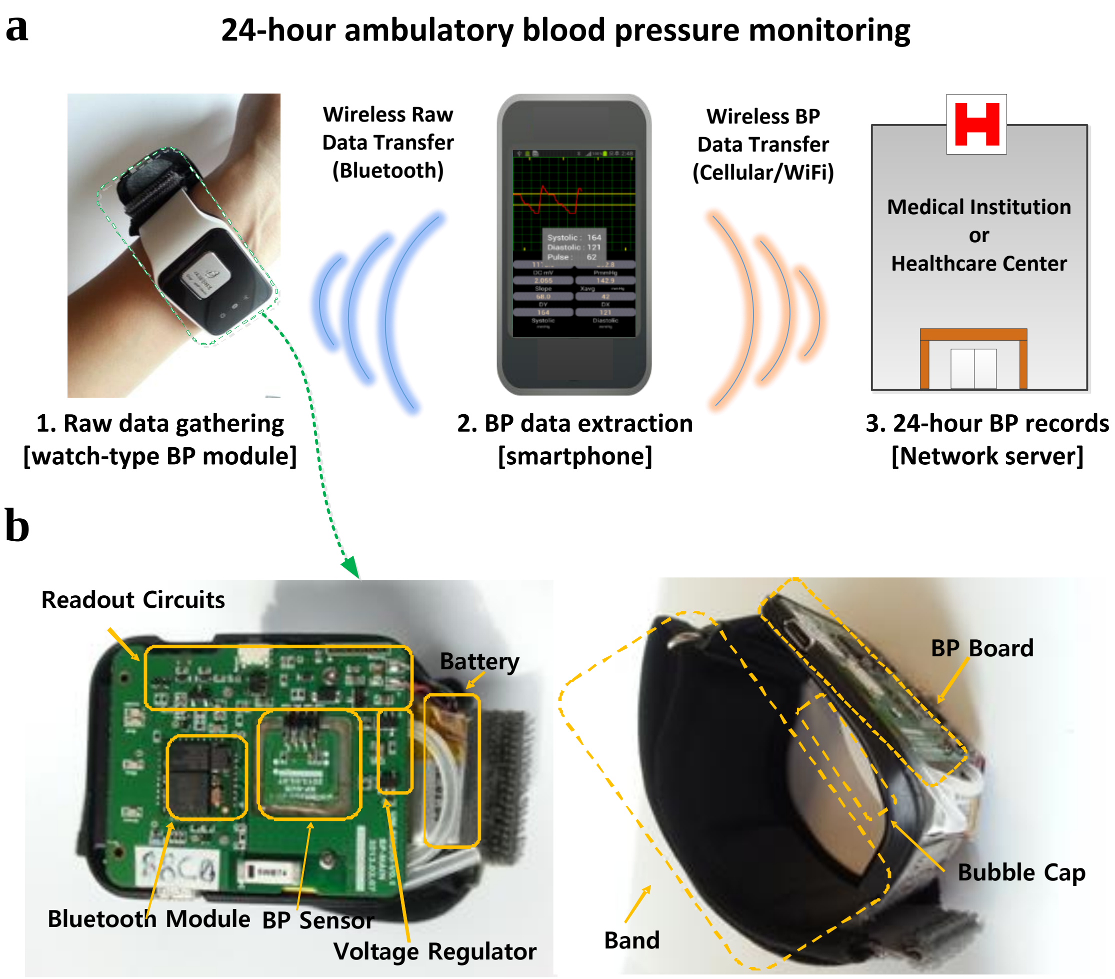

Supplementary Figure S2 | Prototype implementation of continuous non-invasive ABPM. (a) System composition and service scheme of 24-hour continuous ABPM. A watch-type module continuously measures a radial pulse pressure and sends an electric signal to a smart phone, where signal processing is performed to extract the absolute blood pressure. (b) Prototype implementation of a watch-type blood-pressure measurement device which is mainly composed of a pressure sensor (Freescale MP3H6115A), its readout circuits, and a Bluetooth module for wireless connectivity with a smart phone. A full-custom bubble cap is inserted between the module and the skin beyond the radial artery, facilitating comfortable use and also improving measurement efficiency. (All photographs were taken by the authors.)

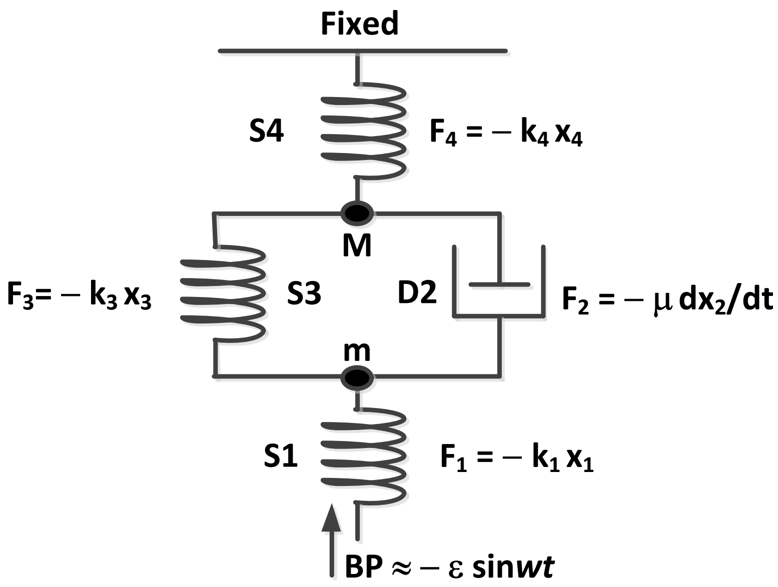

Supplementary Figure S3 | A schematic of spring and dashpot model for the radial artery blood pressure measurement. Compressive displacement of each spring (x) gives the force equation of $-kx$ according to the Hook's law, and a dashpot gives the damping force of $-\mu\, dx/dt$.

Supplementary Methods

Mathematical analysis of spring and dashpot model. This mathematical work to analyze the radial-artery blood-pressure measurement through a spring and dashpot model is based on a previous report of Ref. 17 which originally tried to model the tonometer behavior. In Fig. S3, the spring and dashpot model is redrawn to include force descriptions. For simple analysis, the measurement behavior is considered as motion between two rigid plates. The upper plate for the pressure sensor was assumed to be fixed and the lower plate is movable to apply the arterial blood pressure (BP) which is approximated by a sinusoidal function $-\varepsilon \sin wt$ for convenient analysis. The force-balance conditions at each mass point give the following equations

$$m\left(\frac{d^2x_4}{dt^2}+\frac{d^2x_2}{dt^2}\right)=-\mu\frac{dx_2}{dt}-k_3x_3+k_1x_1 \quad (1)$$

$$M\frac{d^2x_4}{dt^2}=-k_4x_4+k_3x_3+\mu_2\frac{dx_2}{dt} \quad (2)$$

$$-\varepsilon \sin wt=-k_1x_1 \quad (3)$$

The parallel configuration of D2 and S3 gives $x_2=x_3$. For simplicity, the initial conditions of every displacement and its derivative at t=0 are chosen to be zero. According to Ref. 17, if the system (1)-(3) is solved numerically and the k_3 increases from 1 to 10 with time delay, and the transient response of the measurement starts to track the original blood pressure waveform. This means that higher k_3 gives better transfer characteristic until it reaches a saturation limit. Therefore, if this result is combined with the experimental phenomenon in Fig. 2, the k_3 value is directly related to the average BP of X_{avg} and the slope of the ΔY-P_{press} curve where the k_3 varies from 2 to 6 under parameter setup of k_1=1, k_4=5000 and μ_2=1.
